# Health Literacy and People with Intellectual Disabilities: What We Know, What We Do Not Know, and What We Need: A Theoretical Discourse

**DOI:** 10.3390/ijerph16030463

**Published:** 2019-02-05

**Authors:** Cornelia Geukes, Janine Bröder, Änne-Dörte Latteck

**Affiliations:** 1 Department of Nursing and Health, Bielefeld University of Applied Sciences, Interaktion 1, 33619 Bielefeld, Germany; aenne-doerte.latteck@fh-bielefeld.de; 2 Faculty of Educational Science, Bielefeld University, Konsequenz 41A, 33619 Bielefeld, Germany; janine.broeder@uni-bielefeld.de

**Keywords:** health literacy, intellectual disabilities, decision-making, health information

## Abstract

Although health literacy is widely discussed and many heterogeneous conceptualizations exist, people with intellectual disabilities have remained largely unconsidered. The purpose of this conceptual paper is to analyze the particularities of this target group and discuss and consider implications that arise when conceptualizing the health literacy of people with intellectual disabilities. Therefore, we explore relevant approaches from multiple disciplines and examine their transferability to a conceptual understanding of health literacy for people with intellectual disabilities. For future directions we identified three main dimensions: (1) disentangle health literacy from empowerment; (2) apply a positive, asset-based focus to health literacy; and (3) focus on health literacy as a distributed resource across individuals and their individual life-world.

## 1. Introduction

Health literacy has received much attention at the research, practice and policy level. In Germany, a national action plan was endorsed under chairmanship from the national Ministry of Health in spring 2018 [1]. However, decades of developing the health literacy concept has largely overlooked people with intellectual disabilities. In the following, we discuss the urgent need for regarding the specific needs of people with intellectual disabilities in current health literacy research and debates.

This theoretical discourse has two goals: First, we want to show that a target group-specific health literacy concept is urgently needed to counter health inequalities. Second, that health literacy research can benefit from the inclusion of this special group. Furthermore, we want to highlight approaches and concepts that can further enrich future research on health literacy of people with intellectual disabilities, emphasizing the need to consider the specific characteristics of this target group. In the following, a synthesis on the common health literacy concepts and available evidence is presented. A special emphasis is put on how health literacy is currently implemented in practice and which successes have been achieved in the target group. The paper also discusses what is missing in the development of a specific health literacy concept for people with intellectual disabilities.

## 2. What We *Do* Know

In order to give an overview of what is already known about the topic, we will present two popular and much-cited models of health literacy, then we will cite the current research evidence for health literacy among people with intellectual disabilities and focus on them and their relation to health-related behavior and decision-making.

### 2.1. Health Literacy

Health literacy was first mentioned in the 1970s as the ability to understand written health information [2]. This perspective is based on basic literacy skills and has been superseded in recent decades by a multidimensional understanding, namely the combination of different skills needed to access, understand, appraise, and apply health-related information from multimodal sources [3]. Nevertheless, there is a lack of a common definition as well as a consensus model. With regard to outcomes, persons with high health literacy are less often admitted to hospitals, have a higher health status, make better health-related decisions (i.e., decisions more beneficial to their health), and manage their chronic diseases better than people with low health literacy [4,5]. Based on this evidence, it is suggested that health literacy is a determinant of personal health [6]. At the same time—and this already suggests the complexity of this concept—health literacy itself is also influenced by different determinants, namely social, situational, and personal aspects like age, education, culture, language, and educational systems [3]. Moreover, it develops and varies throughout the life course. According to Parker [7], it can be considered a relational construct, namely the product of individual skills and situational or contextual demands that are faced by the individual. Whether a person can make use of their health literacy strongly depends on the specific situation and the contextual factors (such as the level of difficulty of the language or the complexity of the information). This includes the demands that are posed upon the individual within a particular context. In addition, it has been argued that health literacy acts as a mediator between an individual’s social and personal factors and individual health-related outcomes [8]. The confident handling of health-related information also influences individual empowerment. Empowerment is a concept which is taken up within socio-political discussions. As a result, the debate on the concept of health literacy also has an impact on the socio-political level, which will be described in more detail below.

### 2.2. Common Health Literacy Understandings

There are some much cited models in health literacy research. One popular model is the multi-dimensional model used for the large European Health Literacy Survey [3]. It is based on individual skills (motivation, knowledge, competencies) that influence the access, understanding, appraising, and applying of health-related information, which itself is influenced by social and personal determinants.

The model by Sørensen et al. [3] shows how health literacy is applicable to the various levels of health care, disease prevention, and health promotion. It is stated that health literacy has a positive impact for the use of health services, personal health behavior and health outcomes, the subject’s participation in health-related decisions, and the equity that exists within a society, as well as the societal health costs. The model integrates an individual as well as a social level, just as it reflects health literacy over the lifespan.

Another model captures a different perspective: Nutbeam’s tripartite model [9] illustrates the relationship between literacy, health education, and health literacy in three different dimensions and, portrays health literacy as an outcome of health education. The dimension of *functional health literacy* refers to the transfer of health information to improve knowledge and strengthen the participation within health programs. Nevertheless, the functional dimension is often reduced to functional reading, writing, and numeracy skills. The dimension of *interactive health literacy* refers to the development of personal skills in a supportive environment. An individual at this level is able to act independently on knowledge, which enhances motivation and self-confidence. The third dimension is *critical health literacy*. This is based on personal and community empowerment, which is used to be able to contribute to the community and to achieve healthy policies [9].

### 2.3. People with Intellectual Disabilities—A Special Target Group for Health Literacy

As indicated above, numerous studies investigate the outcomes of specific health literacy levels. Still, there is a lack of scientific health literacy studies, which evaluate the group of people with intellectual disabilities [10]. It is estimated that there are approximately 978 million people worldwide with disabilities. Approximately 1–3% of the global population have an intellectual disability—as many as 200 million people. Due to the lack of definitions and the lack of measuring instruments, the number of people with intellectual disabilities can only be estimated very roughly [11].

People with intellectual disabilities are people with an innate low IQ (below 70) who have an increased need for support due to this reduction in intelligence. They do not suffer from a psychological illness or a physical disability. A health literacy concept for people with intellectual disabilities in addition to one of these diseases would have to be discussed differently.

The group of people with intellectual disabilities is at an increased risk of chronic diseases, cardiovascular diseases, and obesity [12]. Additionally, the life expectancy of people with intellectual disabilities increased in the last decades, and hence, an increased presence for age-related diseases in this group can be verified [12]. There is also evidence that people with intellectual disabilities tend to experience specific health problems like incontinence, difficulty swallowing, sensory loss, and adaptability losses [13], as well as oral motor problems and dental problems, fractures, fatigue, arthritis, musculoskeletal deformities, decreased walking ability, progressive cervical degeneration, and a higher risk of developing osteoporosis [14]. Due to the high risk for diseases and chronical illness described above, people with intellectual disabilities have a particularly high need for support and care [12,15]. Evidence from curative education and nursing research shows that people with intellectual disabilities have major problems in the field of language [16]. Particularly, a reduced understanding of speech and a reduced ability to communicate are stated. A reduced ability to communicate is characterized by short and incomplete sentences and altered semantics [17,18]. It has long been known that this has an impact on the access to information and leads to a lack of health-related knowledge [19]. These issues cause problematic access to understandable health-related information and cause difficulties with regard to self-directed decision-making in health promotion and disease prevention in people with intellectual disabilities [20,21]. For these reasons, high sensitivity of caregivers is required. So far, caregivers play a major role in health-related decision-making [22]. However, nurses or caregivers are usually not trained to provide understandable health-related information in a corresponding manner. These aspects indicate that people with intellectual disabilities are a vulnerable group in terms of health, health literacy, health care, and everyday life support. Therefore, it is urgently necessary to include these people in the discussion about health literacy.

The concept of health literacy influences various levels. At a political level, the United Nations Convention for the Rights of People with Disabilities [23] (UN-CRPD) demands empowerment in the health sector based on informed self-determination. As already described, we assume that empowerment is related to health literacy and consider whether it can be strengthened by targeting health literacy in this group (at in the individual and also within professionals, among others health service providers, and care givers). Moreover, health literacy is also formulated as a political goal in national action planning [1]. At the individual level, strengthened health literacy could lead to more informed choices and could have a lasting impact on compliance and health care provision for people with intellectual disabilities [24]. At the social level, the strengthening of health literacy and empowerment could address the inequalities of people with intellectual disabilities [25] (It has to be discussed to what extent a specific health literacy concept for people with intellectual disabilities actually leads to health equality, or on the contrary, leads to increased health differences due to a lack of comparability. This should be an urgent part of future research.). These represent manifold arguments why health literacy is highly relevant for people with intellectual disabilities and why strengthening it is crucial for targeting health inequalities in this vulnerable group. Nevertheless, due to the particularities of people with intellectual disabilities described above, we question whether the application of common health literacy understanding to people with intellectual disabilities without further consideration or testing is feasible. Moreover, there is a lack of scientific evidence for a target group-specific conception of health literacy within this group. Afterwards, further scientific studies could be carried out to investigate expected health literacy outcomes such as decision-making or the effects on health equality. Therefore, scientific knowledge must be generated that takes into account people with intellectual disabilities in their particularities.

## 3. What We *Do Not* Know

With regard to the two multidimensional models by Nutbeam [9] and Sørensen et al. [3], there is a lack of research that provides information regarding whether and how these are applicable for people with intellectual disabilities. Therefore, we still need to discuss and elaborate whether health literacy is a feasible concept for this target group for being empowered in their health-related knowledge and thus participate in health-related decisions.

As there is a scientific gap regarding the health-related knowledge and competencies of people with intellectual disabilities, no statement can be made about how they handle health-related information [26]. There is a lack of insights into key aspects of accessing and using information that takes into account the particularities of people with intellectual disabilities. Neither do we know about the concept of lifelong learning in people with intellectual disabilities and health. Therefore, although the model of Sørensen et al. [3] is relational and multi-dimensional, it remains uncertain whether it is applicable to people with intellectual disabilities due to gaps in the available evidence.

Up to this point, health literacy research that has targeted people with intellectual disabilities has mostly focused on functional health literacy dimensions or on increasing health-related knowledge through the provision of information [25]. Among others, health literacy is frequently used as a synonym with and measured by health-related knowledge or equated with knowledge and information [10]. Scott and Haverkamp [27] have shown in their systematic review that many programs seek to increase the health-related knowledge of people with intellectual disabilities. However, it is difficult to assess the actual effect and impact of these interventions due to the insufficient methodological quality of the quantitative assessment instruments that were used [27]. Most of the available health promotion and health care programs for people with intellectual disabilities aim to enable the individual to better take care of his or her health. Prominently, the underlying assumption is that this can be achieved by focusing on the provision of health information that is easy to understand. Therefore, health-related information is translated into easy-to-read language [28]. By applying a functional health literacy perspective and mostly failing to aim at the special characteristics and needs of people with intellectual disabilities [25], those programs’ positive impact is small and only short-term if using an easy-to-read language within those programs. While research on health literacy with other target groups already considers this and incorporates action dimensions and competences, most research on people with intellectual disabilities, however, continues to focus on functional literacy dimensions.

The dimension of communicative health literacy is rarely represented in the scientific literature. This is, however, crucial as people with intellectual disabilities and their representatives repeatedly criticize the communication behavior of health professionals towards them [29]. Nutbeam’s critical health literacy could play a major role in people with intellectual disabilities, but it is not discussed in the literature at all with one exception: Chinn [25] states that critical health literacy has the potential to integrate an individual’s power and takes into account the linguistic features of the target group. Targeting critical health literacy also has the potential to renegotiate the patients’ role and emerge from paternalistic structures within the contact with health professionals. An important step on the way towards empowerment.

In summary, health literacy with regards to people with intellectual disabilities is often limited to a focus on individual aspects than a holistic concept. In the meantime, and especially with regard to the models described above, we know that health literacy is more than health-related knowledge or access to information. Therefore, we need to move beyond the assumption that the provision of information and the increase of knowledge will automatically lead to better health literacy in the target group and even better health outcomes.

### Underrecognising the Complexity of Health Literacy

From health literacy research with other target groups (e.g., chronic diseases and migrants), we know that there is a wealth of health-related information available, but that the crucial challenge is to filter and judge this information [30]. Above all, quality-assured information is not very accessible. Unfortunately, programs very rarely focus on promoting competencies rather than relying on strengthen knowledge through the provision of written language information [10].

To target this issue, there are clinical studies evaluating training programs for people with intellectual disabilities. These programs are based on visual instructions and question answering in a game format [20] to increase control over the individual’s health and health-related decisions among people with intellectual disabilities. However, this type of intervention requires a lot of time and material. In addition, it is often only accessible to people with a very light intellectual disability or restriction, hence, still excluding people with more severe degrees of disability. This is crucial as it can be assumed that people with a lesser degree of intellectual disability generally possess higher levels of health-related knowledge anyway when compared to the average level in the target group. Hence, it is crucial to recognize the heterogeneity of the target group that requires support structures that are tailored to the person’s individual characteristics and situation.

Literature analyses show that successful communication between health professionals and people with intellectual disabilities is hampered by communicative and organizational deficits and the ignorance of health professionals and support staff [27]. Responding to health needs often requires the knowledge of care givers and the understanding of individuals by health professionals [31]. In addition, scientific research that interviews people with intellectual disabilities as users and captures their subjective perspective is also very rare.

Recently, more and more offices for understandable and accessible language (in Germany *offices for easy language*) have been opened with the goal of translating complex and difficult language into understandable language. Easy-to-read information is, at least in Germany, now widely used and several guidelines have been developed. However, little is known about the effectiveness of easy-to-read resources and the specific components that make them effective. Apart from the fact that this approach is practical, it must however be questioned as to whether it is profitable for the group of people with intellectual disabilities as well.

Until today, there is no clear evidence that introducing accessible information leads to better health outcomes [19,32]. No evidence concludes that adapting the information, which is reliably easier to understand for people with intellectual disabilities than a non-adapted version, leads to increased knowledge or changed health behavior. Furthermore, it is still unclear whether and how personal empowerment and perception of power structures and relations can be influenced through adapting information to an easy-language [25]. In order to establish evidence on whether easy-to-read health information is effective, it is necessary to better understand how people with intellectual disabilities use accessible information in their everyday lives and identify the social practices they engage in when dealing with health information.

The few studies available show that people with intellectual disabilities possess a special understanding of their health-related everyday life. Their employment has a very high priority, and the attributed power in certain health-related actions is transferred to others [22]. It is also known that their everyday life is based on special structures of sense-making and relevance that influence a health-related decision-making process [17]. They use a certain grid to embed new knowledge in existing knowledge, which is often characterized by if-then structures that simplify complex health-related relationships [22]. In order to cope with this complex reality, people with intellectual disabilities make use of the supporting persons and care givers to make health-promoting decisions [33].

Obviously much more is needed than raising pure health-related knowledge to empower people with intellectual disabilities to improve their health literacy.

## 4. What We Need

In the following, we suggest how future research should be aligned with findings from evidence and perspectives from relevant fields.

### 4.1. What to Do with the Evidence?

On the one hand, the conceptual perspective of how health literacy is understood for this target group has to be expanded. This includes considering available findings from health literacy research with other groups for developing a conceptual health literacy understanding for people with intellectual disabilities. Among others, some studies examine vulnerable groups within common health literacy concepts [34]. These discuss how they deal with particularities and what findings are used for further health literacy research. For example, health literacy research with people with chronic diseases or children and adolescents shows that it is important to consider the scientific traditions and perspectives of other adjacent disciplines [35,36,37]. On the other hand, it has to be considered that there is the risk of diluting the concept if a target-group-specific concept contains virtually everything. Nevertheless, it still has to be discussed with a close relation to the target group. Interdisciplinary discussions on the subject of health literacy and people with intellectual disabilities should take into account the special information requirements, namely the scientific evidence why previous information concepts have not led to long-term success. In addition, the learning resources of people with intellectual disabilities should be examined more closely so that they can be included in a health literacy debate in the future. Furthermore, research has to focus on whether supporting persons and caregivers constitute an influencing factor or even a determinant within a target group specific health literacy concept. At the same time, examining the health literacy of supporters in order to receive a holistic picture of the health literacy of people with intellectual disabilities could be very important. These considerations show that adjacent perspectives and disciplines have to be considered, but that they must be discussed in different ways. Moreover, these findings could also be relevant for the general health literacy discussion and could feed back into it in order to help advance the general health literacy concept.

### 4.2. Suggestions for Further Directions

In the following, we will offer suggestions for further research efforts, taking into account the considerations of this article.

#### 4.2.1. Disentangle Health Literacy from Empowerment

Previous discussions in the field of health literacy show that the concept of health literacy is mutually connected with empowerment [3,9]. Empowerment is conceived as people’s participation in their own health though increasing the responsibility for a more active role in decision-making. Nevertheless, making health-related decisions in an empowered manner can present a challenge at the same time. In particular, older people with intellectual disabilities who have been institutionalized for a long time are used to receiving support from caregivers when making health-related decisions [22]. As a result, these people have strong support needs that must be ensured in order to make sure that they have understood all information and are able to use it for making a self-determined decision. Importantly, as mentioned above, research and practice first focus on providing information that can be understood, assessed, and applied by people with intellectual disabilities. However, as supporters or caregivers seem to play a significant role, they should be included in information–mediation concepts as well. This highlights the question of how self-determined and empowered a decision really can be for people who rely on caregivers to such a strong degree. Rather, there is a need to discuss whether a health literacy concept for people with intellectual disabilities should be treated separately from the concept of empowerment. Schulz & Nakamoto [38] discuss this in relation to a general health literacy concept and provide examples of the challenges within empowerment, unless it is ensured that people have adequate health literacy. Furthermore, it can be expected that the concept of empowerment will assume different forms for chronic and acute diseases, as well as for life-threatening and less serious conditions. Therefore, it has to be considered whether empowerment is aimed at managing health and being adherent or making self-determined decisions [38]. It may be stated that empowerment and decision-making as a target for research, policy, and practice are of considerable importance to people with intellectual disabilities. Nevertheless, many factors and variables must be considered and discussed in nuanced perspectives. This is especially relevant as the group of people with intellectual disabilities, who are heterogeneous due to the broad variety of restrictions, such as diseases, but also resources and abilities.

#### 4.2.2. Apply a Positive, Asset-Based Focus to Health Literacy

The discussion on health literacy has mostly implied a deficit perspective on individual skills by focusing on what a person lacks or finds difficult to achieve. However, regarding the discussion about a target group-specific health literacy concept for people with intellectual disabilities, a positive approach that focuses on the resources and assets people have could provide an important new perspective. One approach that focuses on a positive, resource-oriented perspective towards health is the salutogenic model of health from Aaron Antonovsky [39,40]. There is a clear connection between health literacy and salutogenesis as they both focus on the development of competence, individual responsibility, and self-determination. The salutogenetic principles should strengthen resources, resilience, and individual coping abilities [39]. Central to this approach remains the fundamental question of how people assess, appraise, and create health as they move on the health–disease continuum and how they deal with the factors that negatively influence their health. Alfred Schütz [41] has developed a basis in his sociological and phenomenological theory of the life-world, which makes it possible to comprehend the patterns of thought and structures of meaning of an individual methodically. Health-related decisions are also based on individual thought patterns and structures of meaning and relevance; accordingly, the life-world in which people with intellectual disabilities live could play a central role [42]. Research has long focused on the study of specific populations (migrants or those with certain diseases, such as diabetes) [30] in terms of health and disease, including their life-world resources. People with intellectual disabilities were not included in these research efforts. Antonovsky explains the different views and considerations on health and illness on the basis of individual resources as well as the psychological environment and life-world of a person. Antonovsky referred to the ability to activate and use these resources for positively influencing their health and well-being as the sense of coherence (SOC). The SOC consists of the comprehensibility, the manageability, and the meaningfulness with which people encounter challenges and burdens. In the context of health literacy for people with intellectual disabilities, this could be of particular relevance as the handling of health- and disease-promoting determinants and the evaluation of those is also related to the concepts of comprehensibility and manageability. As mentioned above, there are no scientific findings on how people with intellectual disabilities evaluate and classify these factors. Hence, whether comprehensibility and manageability have an impact on the health literacy of people with intellectual disabilities remain questions for further research.

#### 4.2.3. Regard Health Literacy as a Distributed Resources

As mentioned above, the caregivers and supporters of people with intellectual disabilities often play an important role in the health-related choices and decisions of people with intellectual disabilities. Therefore, a target-group-specific concept for health literacy should take into account the life-world (in the sense of the sociological construct according to Schütz [41]), as well as the social structures in which the individual is embedded. With the distributed health literacy perspective, Edwards et al. [43] proposes to include the social variables more conceptually, and thus to improve health management, communication, and decision-making. The authors show that health literacy is not only an individual task; within the general population, health-related information and knowledge were shared with friends and family members. This results in a communication structure that makes use of health literacy skills and could lead to behavior and lifestyle changes [43]. Although this study has merely investigated people with long-term diabetes, it provides insights that could be transferred and applied to people with intellectual disabilities. This perspective enables adding another dimension, the dimension of the third person, to a health literacy conceptualization for people with intellectual disabilities. Such an approach entails that health literacy is not only individualistic but also strongly dependent on the social component. In the case of people with intellectual disabilities, this could be a caregiver or supporter.

### 4.3. Goals of Health Literacy Research for People with Intellectual Disabilities

The lack of clinical studies to measure health literacy among those with intellectual abilities may be due to the fact that traditional health literacy measurement tools are not transferable to people with intellectual disabilities. A long-term goal in the field of health literacy research for people with intellectual disabilities must therefore be to develop a measurement tool for assessing and comparing health literacy levels of people with intellectual disabilities. Ultimately, it would also enable comparability of health literacy levels with other commonly used tools for the general population. Such a measurement tool would need to be free of barriers, hence being in an understandable language and short enough to capture the attention and the concentration of the participants. Simultaneously, it must also reflect the full extent of a target group-specific health literacy definition. However, this definition does not yet exist. Therefore, the eminent, medium-term goal should be to develop a definition that considers people with intellectual disabilities with their particularities. As a short-term goal, we aim to start a scientific discussion together with people with intellectual disabilities regarding what could be characteristics of a proper and feasible health literacy conceptualization for this group. This entails that greater knowledge needs to be generated about how people with intellectual disabilities make health-related decisions, how they interact and derive meaning from information, and what matters to them. Next, it is crucial to understand where and how they need help and support in health terms and what resources and assets they already have or can access if needed. These insights should inform the development of a target group tailored health literacy measurement tool, as none of the currently available tools captures the aspects that were mentioned. Hereby, active involvement and participation of people with intellectual disabilities in the development of conceptual foundation and measurement tools seem to be very important and profitable. Numerous studies with participatory approaches show that this type of research is very successful and generates above-average results [44].

It should also be discussed whether there is a need to first gain a better and differentiated understanding of the target group’s health-related life-world before modifying the concept of health literacy.

## 5. Conclusions

Based on the available scientific evidence of people with intellectual disabilities in the field of health and disease, it seems that caregivers and supporters are a very important factor for health-related decision-making and health literacy. A target group specific health literacy concept should be therefore developed in a multi-dimensional, distributed way that considers the health literacy and role of these third persons. Furthermore, specific characteristics, life trajectories, and experiences should be taken into account. Past, sometimes life-long, experiences seem to have a significant influence on the motivation and knowledge acquisition of people with intellectual disabilities and should therefore be considered in a target-group-specific concept as well. At the same time, the health literacy concept for people with intellectual disabilities should not be dominated by specific perspectives, such as functional health literacy, or outdated assumptions, for instance, that the provision of information automatically leads to better health decisions. It is important to keep trying to be close to the individual abilities and life-worlds in order to maximize the benefit to the target group and not follow a one-size-fits-all approach.

Besides the few initial assumptions and considerations that arose from previous research with people with intellectual disabilities, many knowledge gaps remain. Therefore, more research is needed to better understand the target group, as well as the role of health literacy within this group. In addition, it might be helpful to elaborate whether and how scientific findings from research with other vulnerable groups can be adopted to or are partially valid for people with intellectual disabilities as well. In addition, the methodological analysis of data from people with intellectual disabilities should be further developed. In particular, qualitative research lacks appropriate methods to assess the available data satisfactorily for people with intellectual disabilities.
